# Suppression of microRNA159 impacts multiple agronomic traits in rice (*Oryza sativa* L*.*)

**DOI:** 10.1186/s12870-017-1171-7

**Published:** 2017-11-21

**Authors:** Yafan Zhao, Huili Wen, Sachin Teotia, Yanxiu Du, Jing Zhang, Junzhou Li, Hongzheng Sun, Guiliang Tang, Ting Peng, Quanzhi Zhao

**Affiliations:** 1 Collaborative Innovation Center of Henan Grain Crops, Henan Agricultural University, Zhengzhou, 450002 China; 2 Key Laboratory of Rice Biology in Henan Province, Henan Agricultural University, Zhengzhou, 450002 China; 3 Department of Biological Sciences, Michigan Technological University, Houghton, MI 49931 USA

**Keywords:** Rice, miR159, Agronomic traits, Cell cycle, Plant hormone

## Abstract

**Background:**

microRNAs (miRNAs) are important regulators in plant growth and development. miR159 is a conserved miRNA among different plant species and has various functions in plants. Studies on miR159 are mostly done on model plant, *Arabidopsis thaliana*. In rice, studies on miR159 were either based upon genome-wide expression analyses focused upon responses to different nitrogen forms and abiotic stress or upon phenotypic studies of transgenic plants overexpressing its precursor. STTM (Short Tandem Target Mimic) is an effective tool to block the activity of endogenous mature miRNA activity in plant. Therefore, specific roles of miR159 in rice could be explored by down regulating miR159 through STTM.

**Results:**

In this study, expression of mature miR159 was successfully suppressed by STTM which resulted in the increased expressions of its two targets genes, *OsGAMYB and OsGAMYBL1* (GAMYB-LIKE 1). Overall, STTM159 plants exhibited short stature along with smaller organ size and reduction in stem diameter, length of flag leaf, main panicle, spikelet hulls and grain size. Histological analysis of stem, leaf and mature spikelet hull showed the reduced number of small vascular bundles (SVB), less number of small veins (SV) between two big veins (LV) and less cell number in outer parenchyma. Gene Ontology (GO) enrichment analysis of differentially expressed genes between wild type plants and STTM159 transgenic plants showed that genes involved in cell division, auxin, cytokinin (CK) and brassinosteroids (BRs) biosynthesis and signaling are significantly down-regulated in STTM159 plants.

**Conclusion:**

Our data suggests that in rice, miR159 positively regulates organ size, including stem, leaf, and grain size due to the promotion of cell division. Further analysis from the RNA-seq data showed that the decreased cell divisions in STTM159 transgenic plants may result, at least partly from the lower expression of the genes involved in cell cycle and hormone homeostasis, which provides new insights of rice miR159-specific functions.

**Electronic supplementary material:**

The online version of this article (10.1186/s12870-017-1171-7) contains supplementary material, which is available to authorized users.

## Background

Plant microRNAs (miRNAs) are 20–24 nucleotides (nt) long and widely exist as gene regulators. Most miRNAs negatively regulate the target gene expressions by binding to their cognate mRNA sequences, leading to mRNA cleavage or translational repression [1–3]. Recently, miRNAs were also reported to regulate gene expression by controlling gene methylation [4]. Till date, more than 35,000 mature miRNAs, belonging to 223 species, have been reported in miRBase version 21 (http://www.mirbase.org/). In rice, at least 713 mature miRNAs have been identified and reported in miRBase. In addition to the highly conserved miRNAs, many more of new and species-specific miRNAs have been identified in various crops through high-throughput sequencing technology.

Increasing evidence suggested that miRNAs are indispensable in controlling multiple biological processes including organ morphogenesis and responses to plant hormone and environmental stimuli [5–8]. Altering expression of miRNAs can result in multiple visible phenotypes of a plant. Functions of several miRNAs have been studied in rice. Overexpression of OsmiR156 in rice could modify panicle branches and grain size through down regulation of its targets, *OsSPLs* family transcriptional factors, which subsequently resulted in improved grain productivity [9–13]. Enhanced expression of OsmiR397b in rice increased the overall grain yield up to 25% by enlarging grain size and promoting panicle branching [14]. In addition, loss-of-function of rice miR396 brought out multiple inflorescence architectures and increased grain size and yield, partly due to the altering of plant hormone homeostasis, such as auxin and brassinosteroid (BR) [15, 16]. OsmiR1848 regulates *OsCYP51G3* expression and mediates BR biosynthesis to modulate leaf angle and grain size [17]. Most recently, rice miR528 has been shown to play an important role in viral resistance by negatively targeting L-ascorbate oxidase (AO) mRNA through cleavage [18].

Some plant miRNA families have many members, and their mature miRNAs often have multiple target genes having similar complementary sequence. The miR159 family is one of the most ancient and conserved miRNA families among monocot and dicot plants. Expression of miR159 is abundant and widespread in all plant parts. miR159 targets MYB transcription factors in *Arabidopsis thaliana* [19], predominantly *AtMYB33* and *AtMYB65* [20]. The interplay of miR159 and its target MYB genes is involved in the regulation of vegetative growth, flowering time, anther development and seed size in Arabidopsis [20–22]. The miR159–*MYB101* network in *Arabidopsis thaliana* may be important for the modulation of vegetative growth [23]. *mir159a,b* double mutant has pleiotropic morphological defects, including altered growth habits, curled leaves, small siliques and seeds; and these phenotypes could be reversed if *MYB33* was also mutated in the *miR159a,b* double mutant background. Deregulation of miR159 might be linked with leaf curl disease in tomato [24]. Recent reports found that plant miR159 mimic could even inhibit breast cancer cell growth by targeting *TCF7*, a putative mammalian target for miR159 [25].

Till date, reports about miR159 in rice were mostly focused upon genome-wide expression analyses about responses to different nitrogen forms [26] and abiotic stress [27] or upon phenotypic studies by overexpressing its precursor [28]. Functional studies of loss-of-function mutants of miR159 were done in *Arabidopsis* or other plants through genetic mutants or artificial target mimics. For functional studies of miR159 in rice, we suppressed the expression of miR159 through STTM (Short Tandem Target Mimic, denoted as STTM159), which is an effective tool to block endogenous mature miRNA activity in plant [29]. Our results indicate that down regulation of miR159 results in reduced stature, shorter leaf, panicle length and smaller seeds compared to wild type. This phenotype may result from aberrant cell-cycle due to reduced expressions of cell division, and hormone biosynthesis and signaling genes controlled by miR159-regulated gene networks.

## Methods

### Plant materials and growth conditions

All experiments were performed using rice (*Oryza sativa ssp. japonica* cultivar Nipponbare). Wild type and transgenic lines were transplanted in the field under non-stressed conditions at a research farm of Henan Agricultural University, Henan Province, China (34°53′N, 113°35′E, 94 m altitude) during the rice-growing season with normal management and strictly separate measures. Phenotypic data were collected at the seedling stage, heading stage and maturing stage.

### Vectors construction and rice transformation

**Fig. 1:**
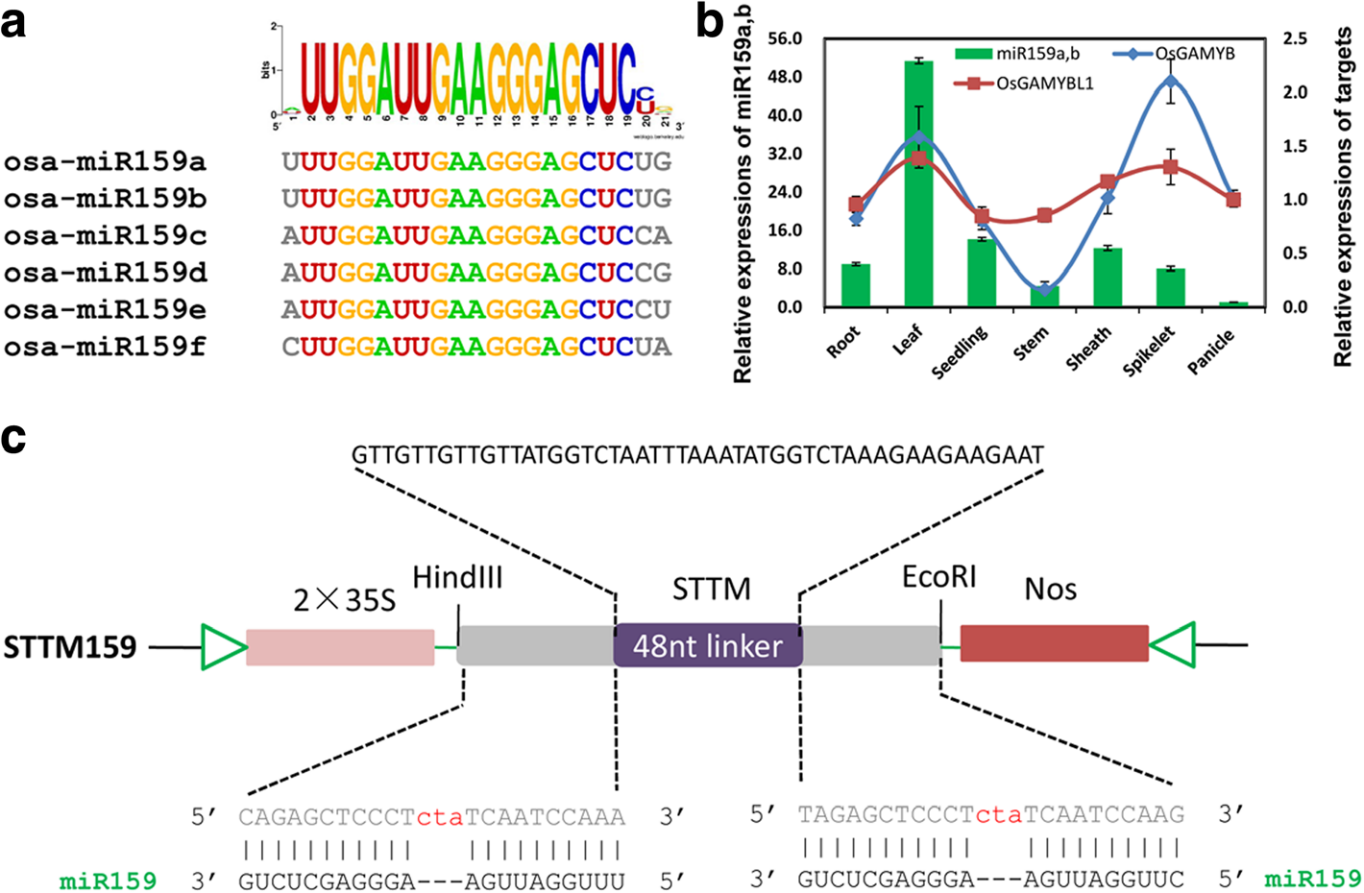
Tissue-specific expression analysis of miR159a and its targets. **a** Sequence alignment of members of OsmiR159 family. **b** Expression patterns of miR159a,b and its two targets during rice growth in various tissues. **c** Schema chart of STTM159 vector construction

For STTM159 suppression vector construction, the fragment with restriction enzyme cutting site HindIII and EcoRI (AAGCTT*TTTGGATTGAAGGGAGCTCTG*GTTGTTGTTGTTATGGTCTAATTTAAATATGGTCTAAAGAAGAAGAATT*TTGGATTGAAGGGAGCTCTG*GAATTC) was synthesized by Sangon Biotech (Shanghai, China) and inserted to the downstream of the 2x35S promoter in *pCAMBIA1301* (Fig. 1c). Subsequent to sequencing, the construct was transformed into *Agrobacterium tumefaciens* strain *EHA105,* and then into rice through Agrobacterium -mediated transformation [30].

### Gene expression analysis

Quantitative real-time polymerase chain reaction (qRT-PCR) was performed to analyze transcript abundance of differentially expressed genes in wild type plants and STTM159 transgenic plants. Total RNA of root, leaf, seedling, stem, spikelet, mature panicle, and developing seeds was extracted by Trizol reagent (Invitrogen) and subjected to reverse transcription with Superscript III Kit (Invitrogen). SYBR Green (Promega, USA) was used as the reporter dye. The primers used are listed in Additional file 1. The mature miR159 level was detected and quantified by a highly sensitive real-time stem-loop qRT-PCR. The reverse transcription reaction was performed with a miR159-specific reverse stem-loop primer (Additional file 1). After stem-loop reverse transcription, miR159-specific forward primer and a universal reverse primer (stem-loop U) were used for SYBR Green-based quantitative RT-PCR. Rice β-actin gene was used as an internal control, and all PCR reactions were repeated three times on three biological replicates.

### Seeds traits measurement

The grain length and width were measured by an automatic seed-size-analyzing system (SC-G, Wanshen, Hangzhou, China). Grain thickness was measured by an automatic Vernier caliper (SATA, Shanghai, China). And 1000 grain weight was measured by 1/10000 balance (Sartorius, Beijing, China).

### Histological analysis

For histological analysis, spikelet hulls, mature flag leaf and the fourth stem (2–3 mm in length) were placed in Formalin-acetic acid-alcohol (FAA) solution (50% alcohol: formalin (37%–40% formaldehyde): glacial acetic acid; 18:1:1) overnight at 4 °C. Then samples were dehydrated in a graded ethanol series (70%, 80%, 90% and 100%), followed by embedding. The samples were dissected and then observed under a light microscope (80I; Nikon, Kanagawa, Japan).

### RNA-seq analysis

Grains of wild type and STTM159 at 6 DAF (days after fertilization) from each plant sample were collected for total RNA extraction. Library construction was conducted with a NEB Next Multiplex Library Prep Set for Illumina (NEB) and then sequenced on an Illumina Hiseq2000 Platform. Number of fragments per kilobase exon per million fragments (FPKM) was calculated before gene expression analysis. Differentially expressed genes whose expression level changed at least 1.5-fold between wild type and STTM159 transgenic spikelets were selected for further GO enrichment analysis. BiNGO was used to analyze item classification of differentially expressed genes. *P*-value of each GO item was calculated by hyper geometric test and was further checked by means of Benjamin & Hochberg. GO item classification including at least five genes and corrected *P*-value < 0.05 was considered as enriched, significantly.

## Results

### Expression profiles of miR159 and its target genes in rice

In miRBase, Osa-miR159 is a conserved miRNA family, and shared conserved 18 nucleotides, from 2 to 19, in rice (Fig. 1a). To determine the expression patterns of miR159, we analyzed the expression of miR159a by stem-loop qRT-PCR. It showed a relative higher expression levels in root, leaf and seedling and a weaker expression levels in stem, spikelet, and mature panicle (Fig. 1b). In rice, miR159 was predicted to target several genes, such as MYBs, and other genes (Additional file 2). According to sequence alignment of the predicted targets with reported known targets (*AtGAMYB33* and *AtAGMYB65*) in *Arabidopsis thaliana*, *LOC_Os01g59660* (*OsGAMYB)* and *LOC_Os06g40330* (*OsGAMYBL1)*, which were also validated in rice (Additional file 3) [26], were selected for further analysis by qRT-PCR using primer pairs spanning cleavage sites. As shown in Fig. 1b, both the targets were found to be expressed lowest in stem, and relatively higher in leaf and spikelet. In general, their expression levels were negatively correlated with the expression level of miR159 except in spikelet (Fig. 1b), suggesting toward a complex relationship between miR159 and its target genes in rice.

### miR159 was suppressed effectively in STTM transgenic plants

**Fig. 2:**
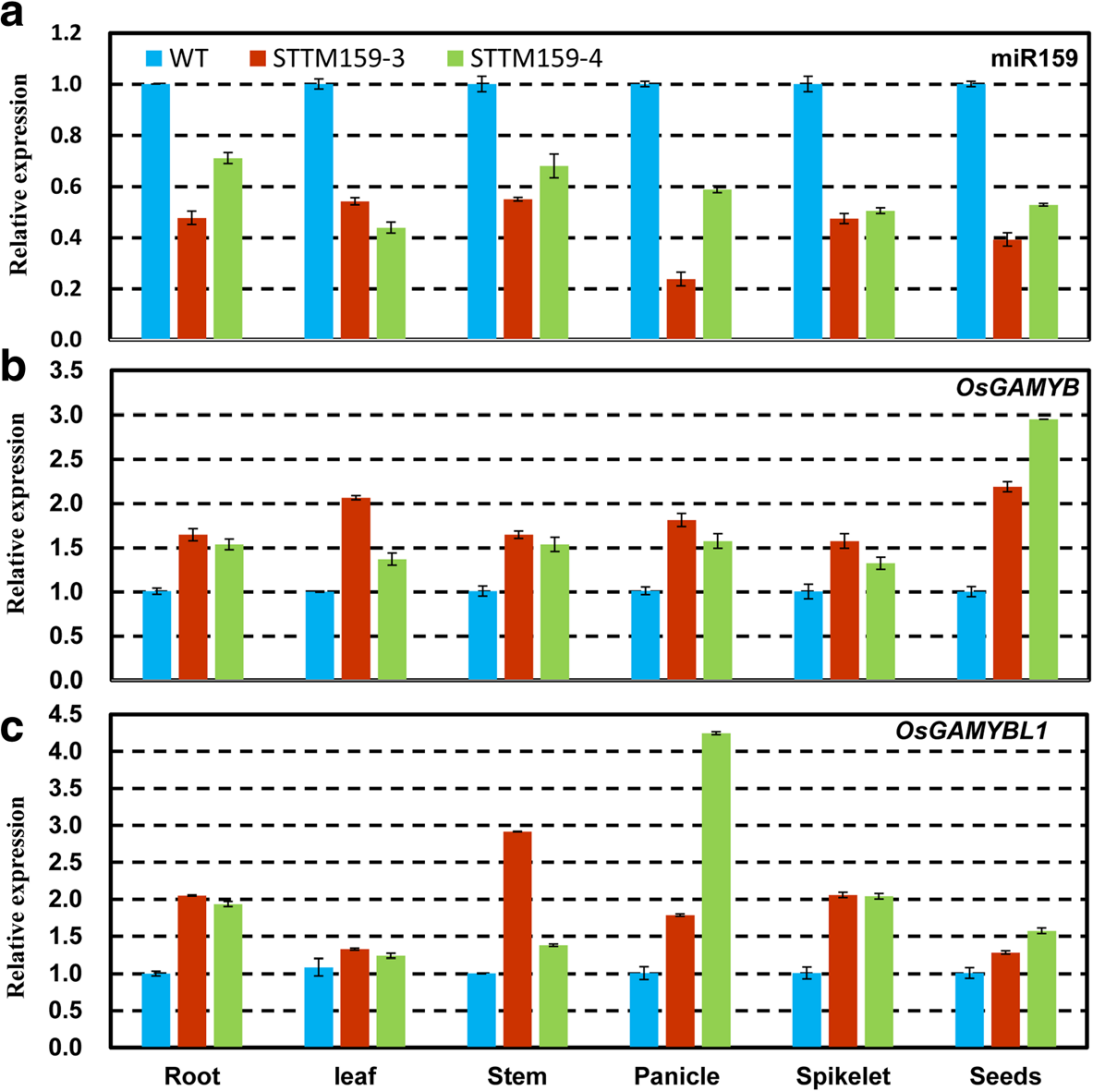
miR159 negatively regulates *OsGAMYB* and *OsGAMYBL1* expressions. **a** Suppressed expression of miR159 in root, leaf, stem, panicle, spikelet and seeds in STTM159 plants. **b-c** Increased expressions of *OsGAMYB* and *OsGAMYBL1* in root, leaf, stem, panicle, spikelet and seeds in STTM159 plants. Experiments were repeated in three independent biological samples and error bars indicate standard deviations of three technical replicates

To evaluate the effects of loss-of-function of OsmiR159 in rice, its expression was suppressed by STTM expressed under 2 × 35S promoter (Fig. 1c). To confirm whether miR159 was down regulated in these transgenic lines, the expression levels of mature miR159 were detected by stem-loop qRT-PCR. As expected, compared with wild type, expressions of mature miR159 were suppressed effectively in root, leaf, stem, panicle, spikelet, and seeds of transgenic rice STTM159 line 3 and 4 (denoted as STTM159–3 and STTM159–4, respectively) (Fig. 2a). These two independent positive transgenic lines were chosen for further phenotypic analysis.

In addition, transcript levels of the two target genes in STTM159 plants were further checked by qRT-PCR. The results indicated that compared to wild type, both the target genes (*OsGAMYB* and *OsGAMYBL1*) showed increased levels in the root, leaf, stem, panicle, spikelet and seeds in STTM159–3 and STTM159–4 transgenic lines (Fig. 2b, c). The significant increase in mRNA levels of the target genes in STTM159 plants suggested toward the regulation of targets transcripts by miR159-mediated cleavage.

### STTM159 reduced plant height and size of leaf, panicle, spikelet and seeds

**Fig. 3:**
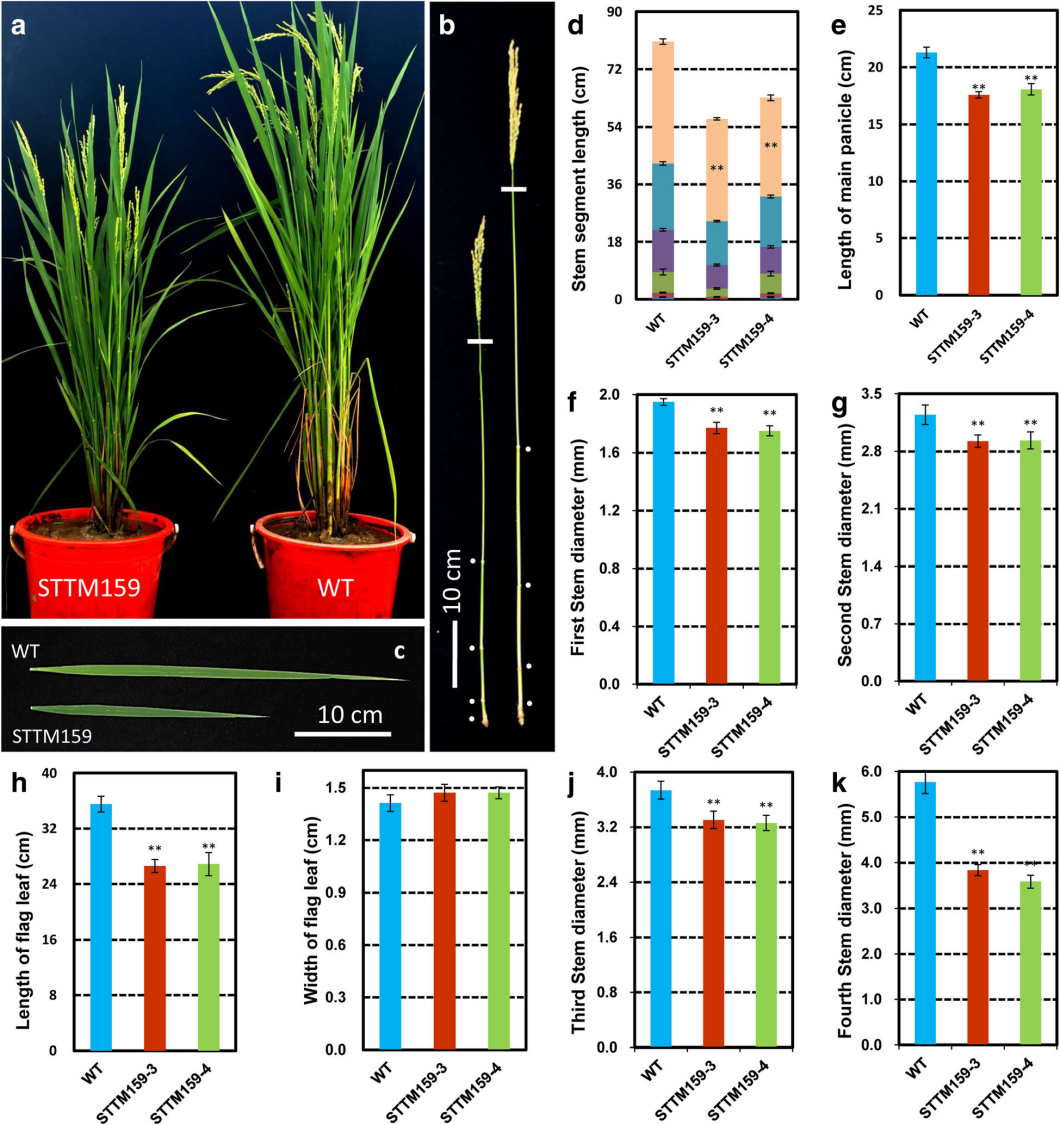
Phenotypes and agronomic traits of STTM159 plants. **a** The whole plant morphology of STTM159 transgenic plants. **b-c** Decreased stem, leaf and panicle length in STTM159 plants. **d-g** Statistical analysis of stem segment length (**d**), length of main panicle (**e**), stem diameter (**f-g**, **j-k**), length of flag leaf (**h**) and width of flag width (**i**). Values are the means ± SD., *n* = 30, **P < 0.01

**Fig. 4:**
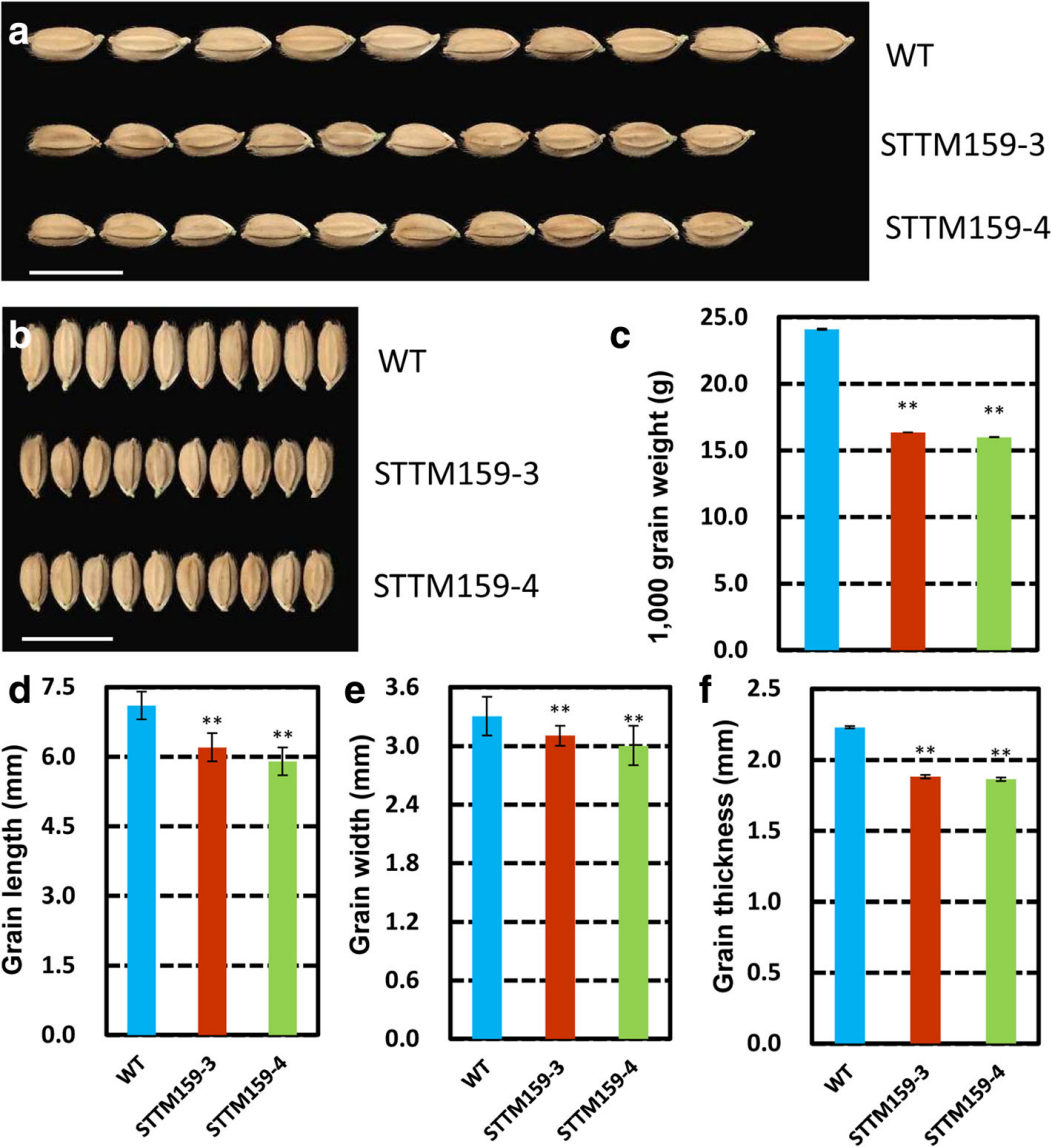
Detailed grain features in STTM159 transgenic plants compared to wild type. **a-b** Grains size comparison of wild type and STTM159 plants. Scale bars, 1 cm. **c** 1000-grain weight of the wild type plants and the STTM159 plants (n = 3 repeats). **d** Grain length; **e** Grain width; **f** Grain thickness, of plants in (**a**) (**d-f**, *n* = 60). Values are the means ± SD., **P < 0.01

STTM159 plants showed morphological defects related to plant stature and organ size. Compared with wild type, STTM159 transgenic plants, most conspicuously showed reduced plant height (Fig. 3a, d), reduced length of main panicle (Fig. 3b, e) and flag leaf (Fig. 3h), and reduced diameter of stems (Fig. 3f–k). Only the width of the flag leaf was found unaltered in STTM159 (Fig. 3i). Statistical data showed that the reduction ranges in the values of plant height, main panicle length, flag leaf length were 23.70%–25.08% (*P* < 0.01), 15.10%–17.37% (*P <* 0.01) and 24.37%–25.14% (*P* < 0.01), respectively. And decreased ranges of each stem segment from first stem segment to the fourth segment were 9.16%–10.18% (*P* < 0.01), 9.62%–9.93% (*P* < 0.01), 11.53%–12.72% (*P* < 0.01) and 33.51%–38.00% (*P* < 0.01), respectively. In addition, other obvious changes resulting from suppression of miR159 were the grain size and weight (Fig. 4a, b). Compared with wild type, grain length (Fig. 4d), width (Fig. 4e) and thickness (Fig. 4f) were reduced by 12.68–16.90%, 6.06–9.09%, 15.51–16.35%, respectively. Subsequently, 1000 grain weight decreased by 32.11–33.58% (Fig. 4c), significantly. Overall, these results suggest that suppression of rice miR159 affected multiple agronomic traits, significantly.

### STTM159 negatively regulates organ size by suppressing cell division

**Fig. 5:**
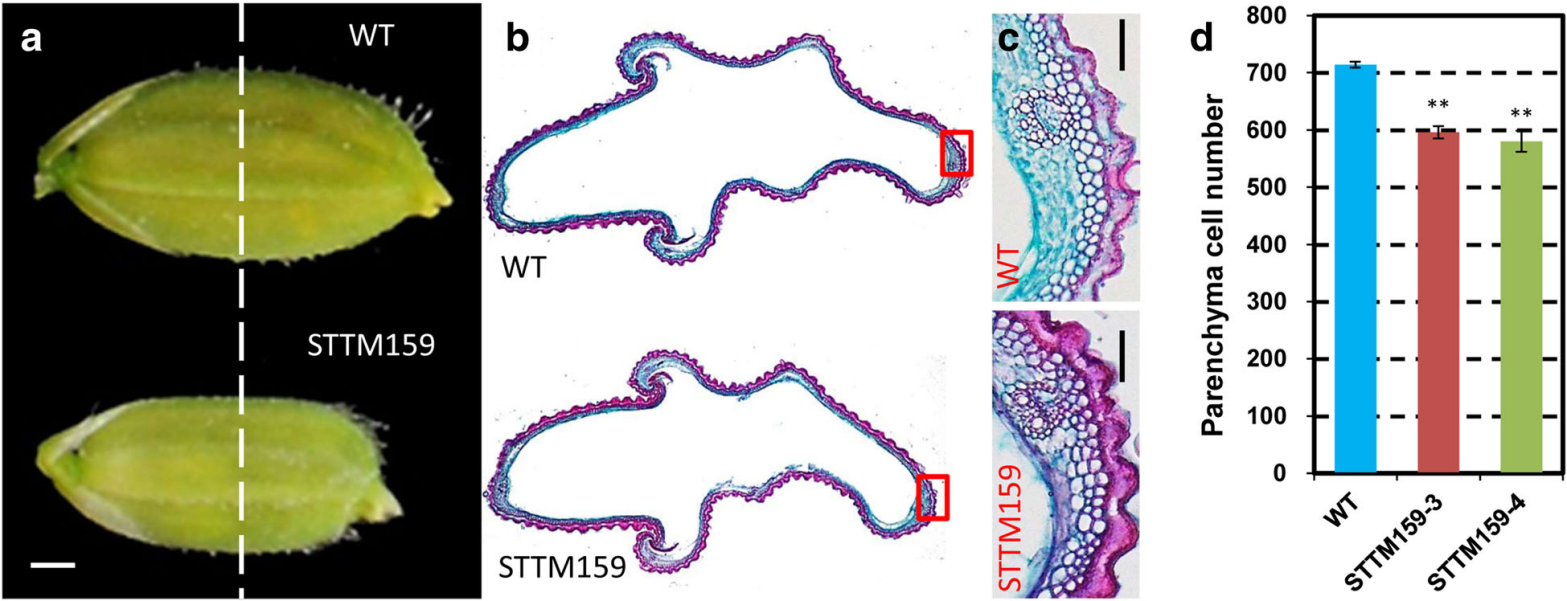
Suppressed expression of miR159 reduces the size of spikelet hulls. **a** Phenotype of mature spikelet in wild type and STTM159 transgenic plants. Dashed line indicates the position of the cross sections. **b** Cross-sections of the spikelet hulls of wild type and STTM159 plants. **c** Magnified views of the cross-section of the spikelet hulls of wild type and STTM159 plants. Scale bars, 1 mm. **d** Statistical data of the cell numbers in the outer parenchyma layer of the spikelet hulls of WT and STTM159 transgenic plants (*n* = 15). Values are the means ± S.D., **P < 0.01

**Fig. 6:**
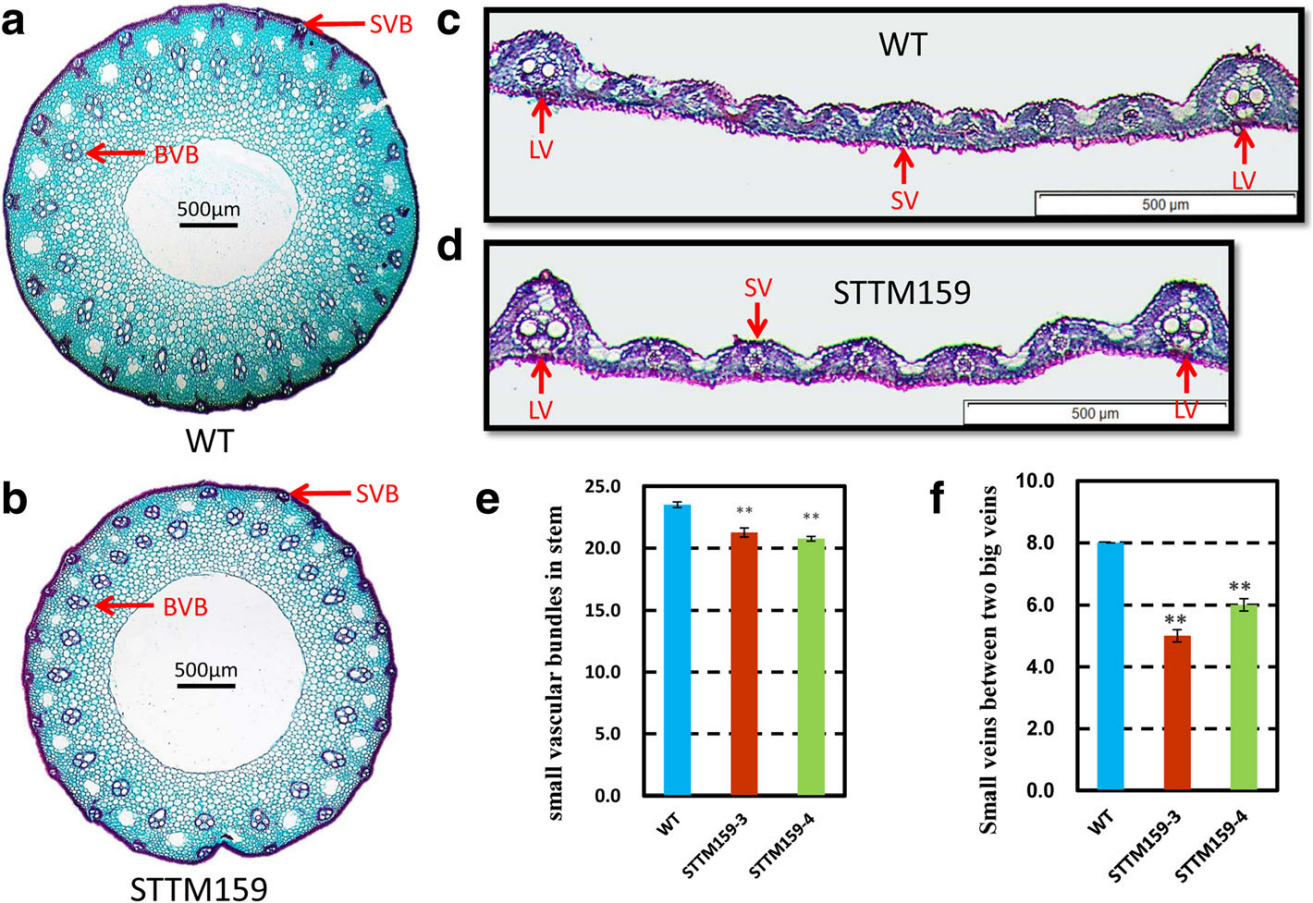
STTM159 plants show reduced stem and flag leaf size. **a-b** Cross-section of the fourth stem in wild type plants (**a**) and STTM159 plants (**b**). BVB, big vascular bundles; SVB, small vascular bundles. Scale bars, 500 μm; **c-d** Transverse sections of the flag leaves in the middle part (between two large veins) of wild type (**c**) and STTM159 plants (**d**). LV, large vein; SV, small vein. Scale bars, 500 μm; **e-f** Statistical data of outer layer vascular bundle in stem (**e**) and small veins between two large veins (**f**) in wild type and STTM159 plants (*n* = 20)

A mature rice grain is mainly made up of a spikelet hull and an endosperm. The size of a grain is restricted by the size of its spikelet hull. As shown in Fig. 5a, we observed a decrease in the length and width of the mature spikelet in STTM159 plants compared to wild type. To confirm the contributions of cell number and cell size to the decreased grain size of STTM159 transgenic plants, histological cross-sections of spikelet hulls were analyzed just before flowering. STTM159 lines had less outer parenchyma cell numbers in the grain hulls (Fig. 5b-d). Furthermore, considering the reduced stem diameter of STTM159 plants, histological analysis of fourth segment of mature stems was done, which revealed that the total number of small vascular bundles (SVB) were decreased significantly in STTM159 transgenic plants compared to wild type plants (Fig. 6a, b, and e). The cell layers in parenchymatous tissue in the fourth stem of wild type plants are significantly more than those in STTM159 plants. Consequently, we see a bigger central aerenchyma in STMM159 stem (Fig. 6a, b). The average numbers of small veins between two large veins were also calculated in wild type and STTM159 transgenic flag leaves. In total, number of small veins (SV) between two large veins (LV) in STTM159 leaves averaged about 6, while that in wild type flag leaves was eight (Fig. 6c, d, and f). Taken together, we conclude that the smaller grain and stem size in STTM159 transgenic plants may result from the suppressed cell proliferation.

### Differentially expressed genes in transgenic STTM159 plants

**Fig. 7:**
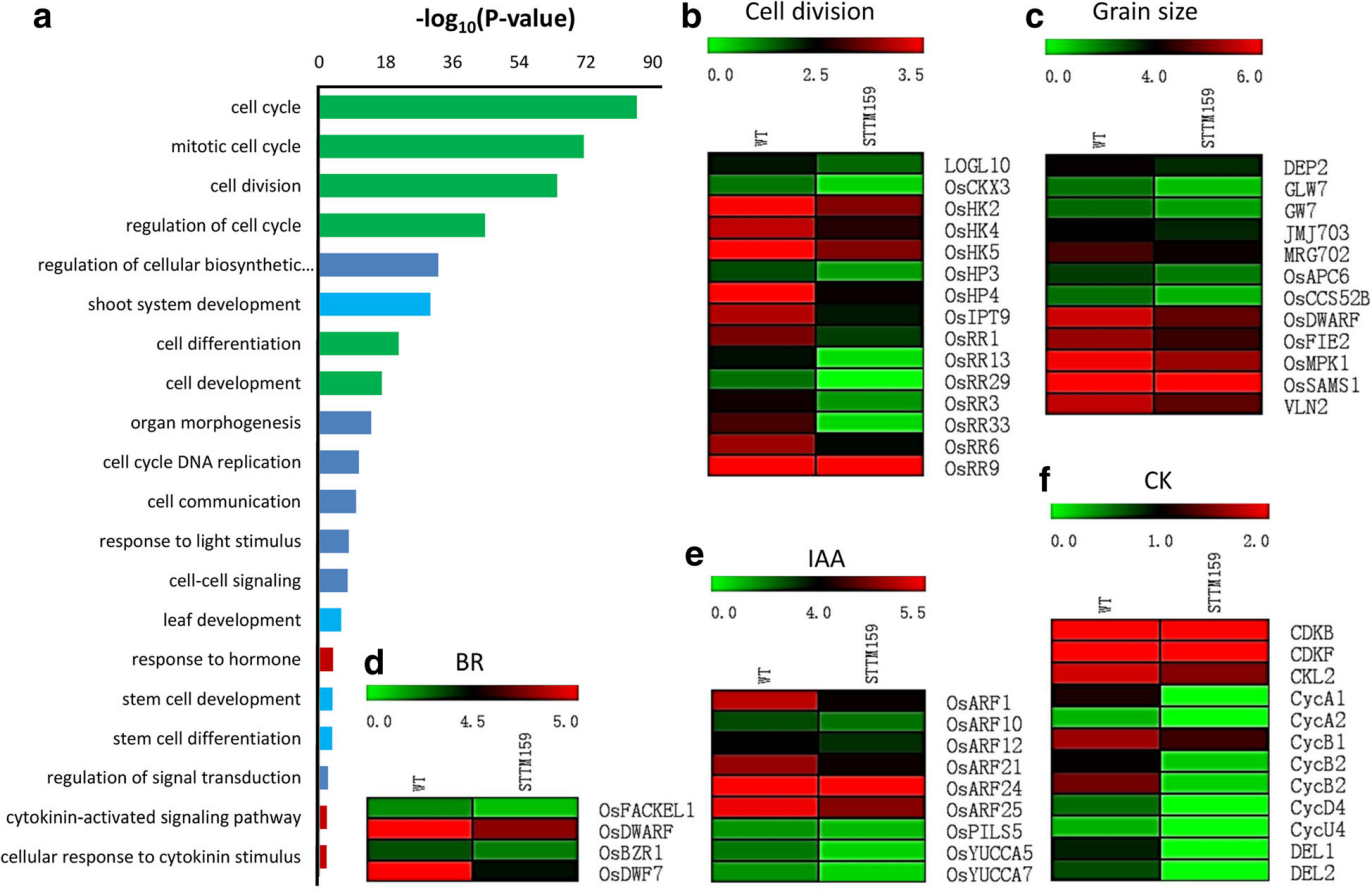
GO enrichment analysis of down-regulated genes in STTM159 plants. **a** Items clustering analysis of the differentially expressed downregulated genes in STTM159 plants compared to WT. Numerical values in the ordinate are the corrected value by -log10. Expressions of genes involved in cell cycle (**b**); grain size QTLs (**c**); BR (**d**); auxin (**e**); CK (**f**) biosynthesis and signaling are decreased in STTM159 plants

**Fig. 8:**
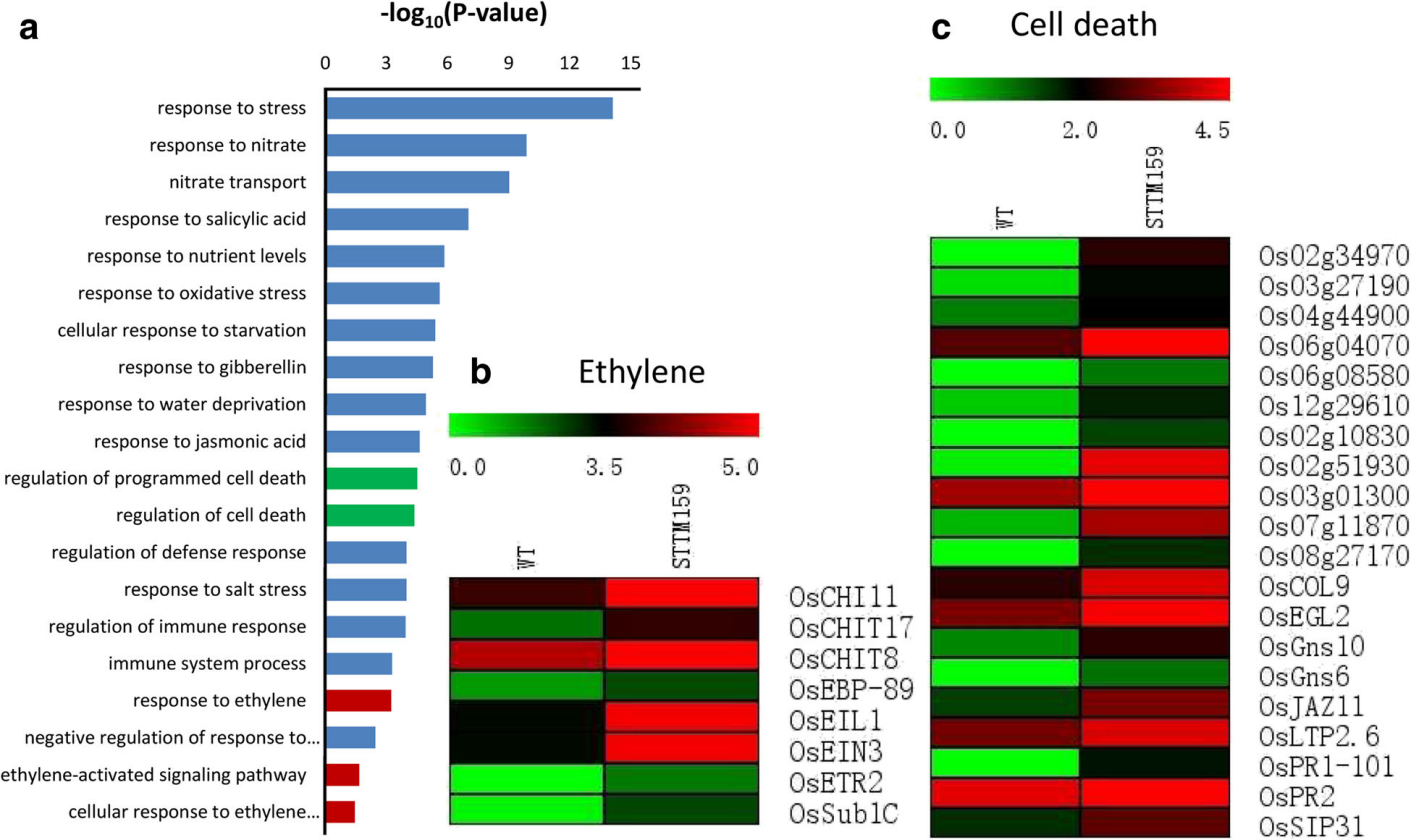
GO enrichment analysis of up-regulated genes in STTM159 plants. **a** Items clustering analysis of the differentially expressed up-regulated genes in STTM159 plants compared to WT. Numerical values in the ordinate are the corrected value by -log10. Expressions of genes involved in response to Ethylene (**b**) and regulation of cell death (**c**) are increased in STTM159 plants

**Fig. 9:**
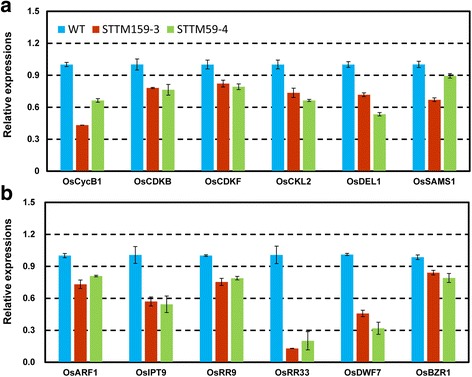
Confirmation of RNA-seq data by real-time PCR. Expressions of some core cell cycle genes and grain size QTL (**a**); and Expressions of some plant hormone biosynthesis and signaling genes (**b**), in wild type and STTM159 plants. All the experiments were performed taking three biological replicates and values are the means ± SD

To further explore the mechanism of osa-miR159 on plant growth and development at genetic level, RNA-sequencing analysis was done using six DAF grains from wild type and STTM159 transgenic plants to compare the global transcriptional profiles of differentially expressed genes between two genotypes. Genes which were changed at least 1.5-fold were identified and subjected to further analysis. In total, 7899 differentially expressed genes were identified in six DAF grains. Such large changes in transcript abundance of genes involved in various pathways implied that STTM159 triggered a change in complex regulatory networks in rice. Detailed statistical data showed that 73.62% of the differentially expressed genes are down-regulated while 26.38% are up-regulated in STTM159 (Additional files 4 and 5). Further GO enrichment analysis of these differentially expressed genes indicated that expressions of genes involved in diverse functional categories were altered in STTM159 plants. The down-regulated genes are involved in pathways related to cell cycle, growth, signal transduction and hormone biosynthesis and signaling, among others (Fig. 7), implying possible contributions of large-scale down-regulated genes to the smaller organ size in STTM159 plants. On the other hand, the prominent categories of the up-regulated genes were related to abiotic and biotic stress and nutrients level response, ethylene response and programmed cell death, among others (Fig. 8). Some of the results of the RNA-seq data of down-regulated genes related to cell cycle and hormone biosynthesis and signaling were verified by qRT-PCR. qRT-PCR confirmed the results of RNA-seq for the selected genes tested (Fig. 9). These results strongly suggest that reduced expression of miR159 suppressed the expression of these genes to restrain cell proliferation to form smaller organs in STTM159 plants.

## Discussion

miR159 is a conserved miRNA among plant species. Rice genome has at least six putative transcriptional units for miR159 precursors according to miRBase 21 (http://www.mirbase.org/). It has been reported that miR159a might be the dominant locus for the production of miR159 [28]. Our study first describes altered agronomic traits of rice under decreased expression of miR159 achieved by STTM technology. In general, STTM159 leads to phenotypic abnormalities including shorter flag leaf, reduced plant height, panicle length, grain size and smaller diameter of stems. In contrast, rice plants overexpressing miR159, showed a severe defect in elongation of the top internode and the panicles developed malformed flowers within the leaf sheaths. Furthermore, the flowers in these transgenic lines showed defects in floral organs and were male sterile [28]. The phenotype of miR159 overexpressing plants was severe compared to that of the *gamyb* mutant, indicating toward the phenotypic contributions of other *GAMYB*-like genes, suppressed in miR159 overexpressing plants [28]. Consistent with this, over expression of wheat miR159 in rice induced male sterility and reduced seed setting rate [31]. However, thousand-fold overexpression of cleavage resistant wheat *TaGAMYB1* gene in rice induced no change in phenotype except that related to flowering time and tiller number [31]. This is not what we observed in STTM159 rice plants, with up- regulated rice *GAMYB* genes. This could possibly be due to combined higher expression of two miR159 targets as opposed to just one in case of *TaGAMYB1* overexpressing lines. Secondly, *OsGAMYBL1* is structurally little different than *OsGAMYB* and *TaGAMYB1* genes, indicating toward additional functions of *OsGAMYBL1*, which could induce new phenotypes when been upregulated in STTM159 plants. Thirdly, some unknown miR159 target genes might contribute to the phenotype of STTM159 plants. In tomato, a non MYB gene (*Solyc12g014120.1*) was found to be a target of miR159, and whose overexpression in tomato reduced the size of leaves and induced formation of abnormal and sterile flowers [32]. *Arabidopsis mir159ab* double mutant had smaller rosettes and formed small siliques with smaller seeds. In contrast, plants expressing its cleavage-resistant target, *AtMYB33,* could phenocopy *mir159ab.* Furthermore, mutations in *myb33* and *myb65* alleles suppressed the *mir159ab* phenotype [20], which implies that deregulation of *AtMYB33* and *AtMYB65* are responsible for *mir159ab* mutant phenotype. But up- or down-regulation of a miRNA may not induce correlated phenotypic changes in plants. Arabidopsis plants overexpressing miR159a/b do not affect leaf development [19], while *mir159ab* double mutants form abnormal leaves that are curled upward [20]. Similarly, overexpressing cleavage-resistant targets such as in the case of *OsGAMYB* or *TaGAMYB1* in rice, may not lead to the similar phenotype as obtained by downregulating miR159. This is because a miRNA usually regulates multiple target genes with either redundant or different functions. In the light of the above studies, it is possible that change in the expression of miR159 targets, *OsGAMYB* and *OsGAMYBL1*, in STTM159 transgenic rice plants, could affect the development of agronomic traits of rice such as plant height, grain size and others, which are not seen in plants overexpressing cleavage resistant targets of miR159. Furthermore, phenotype such as shortened internodes and panicles brought out by suppression of miR159 observed in STTM159, was also observed in the *gamyb* mutant [33], suggesting that other targets regulated by miR159 might also be responsible for the changed agronomic traits. It also suggests that a complex cross-talk between rice miR159 and its downstream targets may exist, whose details should be studied further. It will be interesting to see if manipulation of other miR159-targets could affect agronomic traits as well.

Cell cycle is of vital importance and contributes to histogenesis and organogenesis for spatial and temporal growth and development in plants [34–37]. Regulation of cell cycle is primarily controlled by highly conserved molecular machinery in which cyclin-dependent kinases (CDKs) play a central role [38]. In rice, several regulators of cell cycle have been identified [39]. Mutation in cell cycle genes resulted in normal but overall smaller leaf size [40]. CDKs belong to the core cell cycle genes [41, 42]. In STTM159, most of the core cell cycle genes such as *OsCycB1;1*, *OsCDKF*, *OsCKL2*, *OsDEL1* and *OsCDKB* (a unique class CDKs possessed by plants and had not been described for any other organism), were down-regulated compared with wild type. This indicates that the disruption of cell division in STTM159 transgenic plants could be due to down-regulated expressions of these cell cycle genes (Figs. 7 and 9). It is well known that miRNA has a negative relationship with its downstream targets. Consistent with that, transcripts levels of the two miR159 targets, *OsGAMYB* and *OsGAMYBL1*, were much higher in STTM159 than those in wild type according to our RNA-seq data and results of qRT-PCR (Additional file 2, Fig. 2b-c). It has also been reported that GAMYB contributes to promote programmed cell death (PCD) in *Arabidopsis* [43, 44]. In rice, loss-of-function *gamyb* mutant always shows male sterility, as tapetum degenerates and does not undergo PCD [45]. Furthermore, studies have been reported that reduced cell numbers could be the secondary effect of activated PCD processes [43]. Our results showed that cell numbers in the outer parenchyma layer of the spikelet hulls and number of small vascular bundles (SVB) in stem of STTM159 were much less than those in wild type (Figs. 5 and 6). The RNA-seq data reveals many PCD genes to be up-regulated in STTM159 (Fig. 8). Together with the smaller organs in STTM159 transgenic plants, all these evidences indicate that higher expression level of *OsGAMYB*, because of the suppression of miR159, may contribute to the reduced cell division by activating PCD process in STTM159 transgenic plants. However, the direct evidence to establish this finding should be explored further in the future.

Plant hormones such as auxin, cytokinin (CK), abscisic acid (ABA), ethylene (Eth), gibberellin (GA), jasmonic acid (JA), brassinosteroid (BR) and strigolactone (SL), play important role in plant growth and development [46]. Auxins and CKs are mainly responsible for cell division and expansion, lateral root development, apical dominance, and vascular tissue differentiation [47–49]. Defects in auxin and CK biosynthesis and signaling affect plant organs size. Down-regulation of *OsARF1* (AUXIN RESPONSE FACTOR1) results in shorter leaves and seed size in rice [50], and overexpression of *OsGH3.2* induced dwarfism in rice plants [51]. Our RNA-seq data revealed that expressions of genes related to auxin and CK biosynthesis (*OsYUCCA*, *OsIPT*) and signal transduction (*OsARFs*, *OsRR9* and *OsRR33*) were lower in STTM159 transgenic plants compared to wild type (Additional file 4 and Fig. 7). This may have impaired auxin and CK signaling, resulting in smaller organ size in STTM159 transgenic plants. In addition, auxin activates cell cycle [52] and many cell cycle genes alter their expression level when treated with exogenous auxin or CK. More importantly, cell division is also regulated by some hormones and transcriptional regulation of some core cell cycle genes is controlled by auxin and CK [39, 53]. For instance, CK drives the cell cycle through the induction of D-type cyclins [34] and controls cell cycle at mitosis by stimulating the tyrosine dephosphorylation and activation of p34^cdc2^-like H1 histone kinase [54]. Auxin transcriptionally regulate CDKs inhibitory protein (KRP2) to prevent lateral root initiation by disrupting the G1-to-S transition [55]. Consistent with the viewpoint that gene regulation and the biosynthesis of new proteins are often the consequence of signal transduction, it is very likely that reduced expressions of cell cycle genes in STTM159 were owing to disrupted auxin and CK homeostasis and/or signaling.

In addition to auxin and CK, BRs also mediate many important processes in plant growth and development including cell division and vascular differentiation [56]. In STTM159 plants, expression of many BR synthesis and signaling genes including *OsDWARF*/*CYP85A1* (*brassinosteroid-deficient dwarf1*) and *OsBZR1* were found to be reduced, significantly. Knockdown of *OsBZR1* expression causes reduced seed size and weight by triggering expression of a MYB domain protein, *carbon starved anther* (*csa*) [57]. Our results indicate that genes defective in BR synthesis and signaling could result in dwarfism and reduced grain size in STTM159 rice plants [58]. In addition, BR enhances cell division in the presence of auxin by regulating the expression of cell cycle gene, cyclin D3 [59]. Therefore, we believe that disrupted BR signaling in STTM159 plants might attribute to altered morphological characters, such as plant height in vegetative growth stage and spikelet hull size in reproductive developmental stage. In addition, transcriptional levels of quantitative traits (QTLs), associated with grain size, are reduced significantly in STTM159 transgenic plant compared with WT (Fig. 7c). It is reasonable to speculate that decreased expressional levels of the genes related to grain weight QTLs contributed to the smaller grain size in STTM159 plants.

## Conclusions

The present study indicates that miR159 positively regulates organ size, including stem, leaf, and grain size in rice by promoting cell division. Further analysis of the RNA-seq data indicates that the decreased organ size in STTM159 transgenic plants results from reduced cell divisions, which in turn, may at least partly result from the lower expression of the genes involved in cell cycle and hormone homeostasis. Further studies are required to explore the underlying pathways governed by *OsGAMYB, OsGAMYBL1* and other possible miR159-target genes regulating agronomic traits in rice. Considering that miR159 regulates an important agronomic trait of grain size, manipulation of the expression of miR159 or its target genes can help achieve higher rice yield on account of increased grain size.

## Additional files

Additional file 1: The primers used in the paper. (DOCX 14 kb)

Additional file 2: Predicted targets of OsmiR159 by psRobot and their expressions detected by RNAseq. (DOCX 15 kb)

Additional file 3: Phylogenetic analysis of the targets of *OsmiR159* and *AtmiR159*. (DOCX 54 kb)

Additional file 4: Down-regulated genes in STTM159 detected by RNA-seq. (XLSX 402 kb)

Additional file 5: Up-regulated genes in STTM159 detected by RNA-seq. (XLSX 150 kb)

## Abbreviations

ABA: Abscisic acid
AO: Ascorbate oxidase
BRs: Brassinosteroids
CDKs: Cyclin-dependent kinases
CK: Cytokinin
DAF: Days after fertilization
Eth: Ethylene
FAA: Formalin-acetic acid-alcohol
FPKM: Fragments per kilobase exon per million fragments
GA: Gibberellin
GO: Gene Ontology
JA: Jasmonic acid
OsARF1: Auxin response factor 1
OsBZR1: Brassinazole resistant 1
OsCDKB: Cyclin-dependent kinase B-type
OsCDKF: Cyclin-dependent kinase F
OsCKL2: Cyclin-dependent kinase-like 2
OsCycB1;1: Cyclin B-type 1–1
OsCYP51G3: Cytochrome P450 subfamily 51 G3
OsDEL1: DP-like 1
OsDWARF: brassinosteroid-deficient dwarf 1
OsGAMYB: Gibberellin myb gene
OsGH3.2: Gretchenhagen-3;2
OsIPT: Adenosine phosphate isopentenyltransferase 9
OsRR33: A-TYPE response regulator 33
OsRR9: A-TYPE response regulator 9
OsSPL: Squamosa promoter binding protein-like
OsYUCCA5: YUCCA-like gene 5
PCD: Programmed cell death
qRT-PCR: Quantitative real-time polymerase chain reaction
QTLs: Quantitative traits
SL: Strigolactone
STTM: Short Tandem Target Mimic
TCF7: Wnt signaling transcription factor
