# Supplementary material for: Suppression of microRNA159 impacts multiple agronomic traits in rice (Oryza sativa L.)
Source: BMC Plant Biol. 2017 Nov 21;17:215. doi: 10.1186/s12870-017-1171-7 (PMC5699021; doi:10.1186/s12870-017-1171-7)
Supplement: Supplementary file 1 — The primers used in the paper. (DOCX 14 kb) [file 12870_2017_1171_MOESM1_ESM.docx]

Additional file1. The primers used in this study.

| **Name** | **Sequence** |
| --- | --- |
| miR159a,b_F | ACACTCCAGCTGGGTTTGGATTGAAGGGA |
| miR159a,b_RT | CTCAACTGGTGTCGTGGAGTCGGCAATTCAGTTGAGCAGAGCTC |
| stem_loop U | TGGTGTCGTGGAGTCG |
| OsGAMYB_F | TCCTCGGTGGATCAATTCTC |
| OsGAMYB_R | CTTGAGCCAGCTGTTTGGAT |
| Os06g40330_F | CCATTGCCACACAAGATCAG |
| Os06g40330_R | GATGTCCTAAGGGGTCGTGA |
| OsCycB1;1_F | AATCTCACCGTTCCTACAGC |
| OsCycB1;1_R | AGTAGAGTGCGAGGTAACAAG |
| OsCDKB;2_F | GAGCATCCCTACTTCAACGA |
| OsCDKB;2_R | CAGCATCCAGGAAACAGACA |
| OsCKL2_F | GCAAACTGATGGCAAAGAGG |
| OsCKL2_R | GCCGAGGTTCTGGTTCTCT |
| OsDEL1_F | TATTTCTTACCGCGTGCTT |
| OsDEL1_R | AACAATGAGGCGAGTTTATC |
| OsSAMS1_F | TGAGATTGCTGCTGACCTGA |
| OsSAMS1_R | ATGATGATCTTCCGGCCAGT |
| OsDWARF_F | TGGGCTGCTGAGGAAAACTA |
| OsDWARF_R | ACCTCCTCCAAACAGCATGA |
| OsARF1_F | TCCAAATGCGTCCAAGGTTG |
| OsARF1_R | GGCTTTCATCCGCAGCAATA |
| OsIPT9_F | AGGGTCGACTGCATGATTGA |
| OsIPT9_R | CCGGTTTCCTTCCTTGCAAA |
| OsRR9_F | CCGACATGAGCAAGCTGAAG |
| OsRR9_R | CTGCCTTTCTCTTGTGGCTG |
| OsRR33_F | AGGAAGAGGCAGCTGACAAT |
| OsRR33_R | CATGGAGTCCGACGAGATGA |
| OsDWF7_F | TTTTGCAAGCCATACCGCAT |
| OsDWF7_R | AGTCCATCCACACGGTGTAG |
| OsBZR1/BES1_F | CGTGGAAGGAGAGGGAGAAC |
| OsBZR1/BES1_R | GCTTACATCCCTTGCGGTAG |
