# Supplementary material for: Suppression of microRNA159 impacts multiple agronomic traits in rice (Oryza sativa L.)
Source: BMC Plant Biol. 2017 Nov 21;17:215. doi: 10.1186/s12870-017-1171-7 (PMC5699021; doi:10.1186/s12870-017-1171-7)
Supplement: Supplementary file 2 — Predicted targets of OsmiR159 by psRobot and their expressions detected by RNAseq. (DOCX 15 kb) [file 12870_2017_1171_MOESM2_ESM.docx]

**Additional file 2** Predicted targets of OsmiR159 by psRobot and their expression detected by RNAseq.

| **Gene locus** | **Description** | **Score** | **Expression (FPKM)** | | |
| --- | --- | --- | --- | --- | --- |
|  |  |  | **WT** |  | **STTM159** |
| LOC_Os05g41166 | MYB family transcription factor | 1.5 | 0.01 |  | 0.04 |
| LOC_Os06g40330  (*OsGAMYBL1*) | MYB family transcription factor | 1.8 | 13.14 |  | 20.79 |
| LOC_Os01g59660  (*OsGAMYB*) | MYB family transcription factor | 1.8 | 11.71 |  | 14.87 |
| LOC_Os04g46384 | MYB family transcription factor | 2 | None |  | None |
| LOC_Os03g38210 | MYB family transcription factor | 2 | 8.61 |  | 7.81 |
| LOC_Os05g42240 | hypothetical protein | 2 | 0.32 |  | 0.46 |
| LOC_Os06g46560 | myb-like DNA-binding domain  containing protein | 2.5 | 0.01 |  | 0.06 |
