# Supplementary figures and images for: Suppression of microRNA159 impacts multiple agronomic traits in rice (Oryza sativa L.)
Source: BMC Plant Biol. 2017 Nov 21;17:215. doi: 10.1186/s12870-017-1171-7 (PMC5699021; doi:10.1186/s12870-017-1171-7)

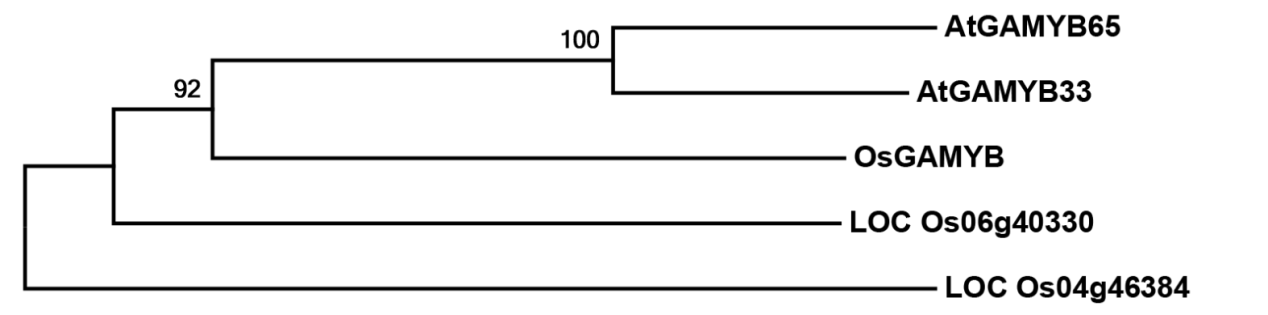


Additional file 3. Phylogenetic analysis of the targets of *OsmiR159* and *AtmiR159*.

Supplement: Supplementary file 3 — Phylogenetic analysis of the targets of OsmiR159 and AtmiR159. (DOCX 54 kb) [file 12870_2017_1171_MOESM3_ESM.docx]
